# Non-pharmacological treatments for schizophrenia in Southeast Europe: An expert survey

**DOI:** 10.1177/00207640211023072

**Published:** 2021-08-14

**Authors:** Lidija Injac Stevović, Selman Repišti, Tamara Radojičić, Norman Sartorius, Sonila Tomori, Alma Džubur Kulenović, Ana Popova, Martina Rojnić Kuzman, Ilias I Vlachos, Shukrije Statovci, Alexei Bandati, Antoni Novotni, Stojan Bajraktarov, Anca-Livia Panfil, Nadja P. Maric, Mirjana Delić, Nikolina Jovanović

**Affiliations:** 1 Faculty of Medicine, University of Montenegro, Podgorica, Montenegro; 2 Psychiatric Clinic, Clinical Center of Montenegro, Podgorica, Montenegro; 3 Association for the Improvement of Mental Health Programmes (AMH), Geneva, Switzerland; 4 University Hospital Center ‘Mother Teresa’, Tirana, Albania; 5 Clinical Center of University of Sarajevo, Sarajevo, Bosnia and Herzegovina; 6 Mental Health Center ‘Prof. Nikola Shipkovenski’, Sofia, Bulgaria; 7 Zagreb School of Medicine, Zagreb University Hospital Center, Zagreb, Croatia; 8 First Psychiatry Department, Eginition Hospital, National and Kapodistrian University of Athens, Athens, Greece; 9 Psychiatry Clinic, Prishtina, Kosovo†; 10 Republican Dispensary of Addictions, Chișinău, Moldova; 11 University Clinic of Psychiatry, Skopje, North Macedonia; 12 Liaison Psychiatry Department, County Emergency Clinical Hospital ‘Pius Brinzeu’ Timisoara, Bucharest, Romania; 13 Faculty of Medicine, University of Belgrade and Institute of Mental Health Belgrade, Serbia; 14 University Psychiatric Clinic, Ljubljana, Slovenia; 15 Queen Mary University of London, London, UK

**Keywords:** Non-pharmacological treatments, schizophrenia, mental health services, expert survey, Southeast European countries, psychosocial interventions

## Abstract

**Background:**

Non-pharmacological treatment for schizophrenia includes educational, psychotherapeutic, social, and physical interventions. Despite growing importance of these interventions in the holistic treatment of individuals with schizophrenia, very little is known about their availability in South-East European countries (SEE).

**Objective:**

To explore mental health care experts’ opinions of the availability of non-pharmacological treatment for people with schizophrenia in SEE.

**Methods:**

An online survey containing 11 questions was completed by one mental health expert from each of the following SEE countries: Albania, Bosnia and Herzegovina (B&H), Bulgaria, Croatia, Greece, Kosovo^†^, Montenegro, Moldova, North Macedonia, Romania, Serbia, and Slovenia. Data were collected on estimated rates of received non-pharmacological interventions, type of services delivering these interventions, and expert views of availability barriers.

**Results:**

In eight countries, the estimated percentage of people with schizophrenia who receive non-pharmacological treatments was below 35%. The primary explanations for the low availability of non-pharmacological treatments were: lack of human and financial resources, lack of training for clinicians, and pharmacotherapy dominance in the treatment for schizophrenia.

**Conclusion:**

Lack of personal and institutional resources and state support were identified as primary obstacles to staff training and delivering non-pharmacological treatments to people with schizophrenia on individual and systemic levels, respectively. This evidence can be used to improve holistic, evidence-based treatment for schizophrenia in the SEE countries.

## Introduction

Schizophrenia is considered a severe and chronic mental illness with complex symptomatology and a prevalence of approximately 1% in the general population (e.g. Stilo et al., 2012). The symptoms usually vary but often include issues with discriminating between what is and is not real; hearing voices; conspiracy theories involving friends and family members, deficits in the social sphere of life (e.g. social withdrawal), as well as difficulty with expression and experiencing emotions (Sadock et al., 2014). Apart from pharmacological therapy, which is the first-line treatment for schizophrenia (American Psychiatric Association, 2010; Patel et al., 2014), the majority of treatment guidelines officially recommend the use of non-pharmacological treatments, which include psychosocial interventions along with various modalities of psychotherapy such as cognitive-behavioral therapy (CBT) and family therapy (e.g. Ventriglio et al., 2020).

In the last two decades we are witnessing a growing interest in non-pharmacological treatment for schizophrenia largely due to inability to achieve complete recovery with antipsychotic medication alone (Leucht et al., 2009) and the high reported rates of medication non-adherence (Lacro et al., 2002). However, non-pharmacological treatment seems to be insufficiently used in clinical practice (e.g. Andreou & Moritz, 2016). There is some evidence that non-pharmacological treatments delivered to people with schizophrenia who were taking minimal doses of antipsychotics or no antipsychotics could have the same positive outcomes as those who take standard/usually prescribed doses antipsychotics (Cooper et al., 2019). Non-pharmacological treatments delivered to individuals diagnosed with schizophrenia focus on wide range of outcomes such as education, explanation, assessment, support, reality reinforcement, building concentration, facilitating relationships and communication, dealing with challenging behavior (e.g., self-harm, aggression), treating non-psychotic symptoms (e.g., mood disturbance, anxiety), increasing skills of people with schizophrenia in their daily life organization, and working with their families (CRAG Working Group on Mental Illness, 1995). Non-pharmacological treatments can help people with schizophrenia as they often encounter issues that include poor self-care, social isolation, unemployment, and service and family dependency (McCann, 2001). In line with that, non-pharmacological treatments of people with schizophrenia have to be designed as a multidimensional approach, aiming at increasing their social competence and reinforcing supportive employment, while, on the other hand, dealing with potential cognitive deficits and negative symptoms (Kopelowicz et al., 2006). Lecomte et al. (2014) reported that there are five evidence-based psychosocial interventions for treating schizophrenia, achieving best effects if combined in the following way: CBT and family psychoeducation, CBT and skills training, CBT and supported employment, supported employment and social skills training and supported employment and cognitive remediation.

Based on their expert survey conducted in Central and Eastern European countries, Winkler et al. (2017) concluded that mental health care services in that region are mostly offered in psychiatric hospitals. According to the mental health policy analysis report for Bosnia and Herzegovina (B&H), Kosovo^†^, Montenegro, North Macedonia, and Serbia, the predominant way of treating people with schizophrenia in these SEE countries includes inpatient care and pharmacotherapy treatment. However, almost no information is available on the availability of non-pharmacological interventions in clinical practice. The present study aims to address this gap in the current knowledge by exploring expert views of the availability of non-pharmacological treatments for people with schizophrenia in 12 SEE countries. For the purpose of this study we defined non-pharmacological treatment as educational, psychotherapeutic, social and physical interventions, excluding alternative therapies.

## Method

### Experts

The expert survey was distributed online to 12 experts, one from each of the following countries: Albania, B&H, Bulgaria, Croatia, Greece, Kosovo^†^, Moldova, Montenegro, North Macedonia, Romania, Serbia, and Slovenia. All experts were psychiatrists involved in clinical and/or research activities with people diagnosed with schizophrenia in the leading psychiatric institution in the country (i.e. a teaching/training institution in its capital city). They were on average, 47.5 years old (*SD* = 8.75) and their average professional experience in the field of mental health equals 19.4 years (*SD* = 7.60). Nine out of 12 participants were females.

### Instrument and procedure

The expert survey was used to gather information on the estimated percentage of people with schizophrenia receiving non-pharmacological treatment in the participating countries; the possible explanation of reported rates; services specialized for delivering non-pharmacological treatments for people with schizophrenia; and settings/services providing non-pharmacological treatments for schizophrenia (inpatient and outpatient services, private practice, and other type of service providing such a mental health care). The expert survey also included sociodemographics (experts’ country, affiliation/institution, age, gender, profession, and years of their professional experience in the field of mental health). For more details on the expert survey, please see Appendix.

The study included experts from leading mental health care institutions in the participating countries. The reason for choosing these institutions was that they are more likely to provide non-pharmacological treatment for people with schizophrenia compared to other institutions in the country. The experts were identified through professional networks in SEE and contacted by email or phone. These experts were contacted based on their extensive knowledge and experience with different types of treatment for schizophrenia. The expert survey was distributed using individual email addresses. The data were collected from the beginning of January 2020 until the end of April 2020.

### Data analysis

Descriptive statistics was used for closed-ended questions (no. 1–7). The summary of answers to the main questions (no. 8–11), have been displayed for each country, separately.

## Results

The estimated percentages of people receiving non-pharmacological treatment in the participating countries ranged from 2% (Romania) to 70% (Albania). Data for each country is shown in Table 1. Experts from Kosovo^†^ and Slovenia felt unable to provide the estimation.

**Table 1.** The estimated percentage of people with schizophrenia receiving non-pharmacological treatment in 12 SEE countries with possible explanations of low availability.

Country	Estimated percentage of people	Explanation
Albania	70%	N/A
B&H	5%	During and after the war in B&H (1991–1995), the number of non-pharmacological services was reduced
		Biological psychiatry is the prevailing doctrine in clinical work
		Poor motivation of students and trainees to study Psychiatry
		Brain drain of health professionals
Bulgaria	20%	These interventions are not financially supported by the National Health Insurance Fund and some people with schizophrenia are not able to pay for them
Croatia	25%	Small number of institutions offer these interventions, for example, three hospitals and two community mental health centers in the whole country
Greece	10%	Pharmacotherapy is a predominant way of treating people with schizophrenia
Kosovo^†^	Difficult to give such an estimate	Stigmatization of people with mental illness
		Lack of information regarding these types of interventions among people with schizophrenia
		Difficult economic situation (lack of financial resources)
		Mental health services are not available locally and therefore difficult to reach by patients
		Low number of clinicians who work with people with schizophrenia
		Lack of coordination between mental health services
Moldova	30%–40% (PIs and PTs are available to all patients with psychotic disorders on their request)	Lack of formal structure and strategy in delivering these interventions.
		Lack of clinicians delivering these interventions
		Heavy clinicians’ workload
Montenegro	20%–30%	The organization of mental health care system is not concordant with contemporary trends in psychiatry
		Lack of financial resources
		Lack of human resources
		Law does not define clearly the delivery of these interventions
		Professional association of psychotherapists does not exist
		Lack of systematic education and training for non-pharmacological treatment for schizophrenia
North Macedonia	20%–30%	The dominance of the pathocentric treatment approach
Romania	<2%	Case management is not clearly structured
		Operational procedures on the national level do not exist.
		Lack of training at public institutions.
Serbia	25%–35%	Lack of training for clinicians
		The dominance of medication-based therapy
Slovenia	Difficult to give such an estimate	The answer has not been elaborated.

The analysis revealed four main reasons for low estimated rates of received non-pharmacological treatment: (1) pharmacotherapy, as recommended in treatment guidelines, is a dominant approach to treatment of schizophrenia in B&H, Greece, North Macedonia, and Serbia, (2) lack of human resources (identified by the experts from B&H, Kosovo^†^, Moldova, and Montenegro), (3) lack of financial resources (Bulgaria, Kosovo^†^, and Montenegro), and (4) lack of training for clinicians (Romania, Serbia, and Montenegro).

Experts were also asked to provide a list of services in their country specialized in delivering non-pharmacological treatment to people with schizophrenia (Table 2).

**Table 2.** Types of services offering non-pharmacological treatment for schizophrenia in 12 SEE countries.

Country	Statutory services		Private sector	Third/voluntary sector
	Inpatient	Outpatient		
Albania	● Non-pharmacological treatment delivered by psychologists and occupational therapists ● Individual and group sessions	● Organized and delivered in CMHCs, in a way similar to that in inpatient units)	● Non-pharmacological offered by psychologists ● Frequency: 1–2 times a week ● Price: 20–40 euros	–
B&H	● Therapeutic community meetings, occupational therapy, and art therapy ● All delivered by inadequately trained clinicians	● Coordinated care services ● Assertive case management	● Not offered for schizophrenia	● Very strong users’ associations (occupational and body therapy)
Bulgaria	● Psychosocial rehabilitation program ● Examples of non-pharmacological treatments: art therapy, group therapy	● Day hospital ● Groups for psychosocial rehabilitation art therapy and outdoors activities (e.g. going to the museum) that usually meet twice a week	● Adherence/compliance therapy ● CBT	–
Croatia	● A special ward for psychosocial interventions for schizophrenia in the psychiatric hospital ‘Sveti Ivan’ ● Psychoeducation, supportive groups, therapeutic community, occupational therapy	● Day hospitals in Zagreb, Rijeka, Slavonski Brod and Rab ● Psychoeducation, social skills training, family therapy, metacognitive training, psychodynamic therapy ● A small number of patients treated in CMHC	● Very rarely ● Dependent of the clinician’s experience	–
Greece	● Ergotherapy starts during hospitalization whereas family therapy and psychoeducation start after discharge	● Non-pharmacological treatment offered by: University Mental Health Research Institute, Center for Psychotherapies, and Day Care Hospital of Vironas	● Many psychiatrists offer non-pharmacological treatments as part of their private practice	–
Kosovo^†^	● Individual and group non-pharmacological treatment ● A separate department for non-pharmacological treatment does not exist ● There is a special time in the schedule for non-pharmacological treatment delivery	● A separate department for non-pharmacological treatment does not exist ● Better organized in CMHCs and offered in both individual and group form.	● Non-pharmacological treatment offered only in individual form	–
Moldova	● Non-pharmacological treatment delivered at the Department of Psychiatry ● Non-pharmacological treatment mostly offered by clinical psychologists ● Planned during the case management discussion ● Special time slots dedicated to individual and group therapy	● Delivered in CMHCs by psychologists and social workers ● Mostly individual interventions ● In rural areas: mobile teams as facilitators of their delivery	● Delivered by psychologists through their private practice (CBT, psychoeducation, and Neuro-Linguistic Programming – NLP)	–
Montenegro	● Family and psychoanalytical therapy ● Therapeutic community meetings ● Non-pharmacological treatments delivered at Psychiatric Clinic during regular sessions	● Outpatient clinic within the Psychiatric Clinic ● Nine CMHCs ● Family and psychoanalytical therapy delivered by the same clinicians working in the inpatient unit	● A small number of psychiatrists, psychologists, and psychotherapists deliver non-pharmacological treatment to people with schizophrenia in private practice	–
North Macedonia	● Day Hospital (University Clinic of Psychiatry) ● Non-pharmacological treatment delivered in both individual and group form	● Seven CMHCs ● Non-pharmacological treatment delivered in both individual and group form	● Non-pharmacological treatment for schizophrenia are minor part of treatment in private practice settings.	–
Romania	● Psychologists, in collaboration with psychiatrists, offer support to people with schizophrenia	● Psychologists offer some non-pharmacological treatments in CMHCs ● No department of psychotherapy	● Psychiatrists and patients can freely choose various types of treatment with psychotherapists who are usually self-employed	–
Serbia	● Day hospitals in the university psychiatric institutions	Two leading on the country – Institute of Mental Health in Belgrade and Psychiatric Clinic of the University Clinical Center in Belgrade	● Delivered to people with schizophrenia	● Training in non-pharmacological treatment is organized by NGO
Slovenia	● Specialized psychotherapeutic wards for people with schizophrenia (University Psychiatric Hospital in Ljubljana) ● Psychoeducation, supportive and social skills groups, CBT, psychodynamic therapy, etc. ● Special schedules including group therapy; however, individual sessions are also held, according to the needs of the patient.	● All six psychiatric hospitals provide outpatient treatment ● Usually, non-pharmacological treatments are delivered in individual form while some services include group therapy ● Outpatient services are also available in dispensaries throughout the country ● Delivered by psychologists, psychiatrists, occupational therapists, and social workers	● A network of private outpatient services mostly covered by health insurance ● Non-pharmacological treatments mostly delivered in individual form	● Community services include NGOs and social work centers ● Employment/vocational rehabilitation, educational programs, day centers, group homes, relative groups

In Albania, delivering non-pharmacological treatment is similarly organized in inpatient and outpatient services. They are delivered by psychologists and occupational therapists, in both individual and group form. In addition, they reported private initiatives where they are usually offered by psychologists, one to two times per week. In this country, some NGOs provide training in CBT, psychodynamic therapy, psychodrama, and some other non-pharmacological treatment.

In B&H, non-pharmacological treatments include therapeutic community meetings, occupational therapy, and art therapy. Outpatient services provide coordinated care services and assertive case management. According to the expert’s opinion, the services from the NGOs are delivered by insufficiently trained individuals and volunteers. However, there are very strong users’ associations offering occupational and body therapy.

In Bulgaria, inpatient services include psychosocial rehabilitation program as well as art and group therapy. Outpatient services include day hospital, with groups for psychosocial rehabilitation, art therapy and outdoors activities (e.g. going to the museum). In private practice, adherence/compliance therapy and CBT are offered. Services/institutions for training in non-pharmacological treatments include the Bulgarian Association of CBT, Bulgarian Psychoanalytical Society, Bulgarian Association of Hypnosis and Hypnotherapy, Bulgarian Association for Gestalt therapy, and Institute for Psychodrama.

In Croatia, inpatient service has a special ward for delivering psychosocial interventions (psychoeducation, supportive groups, therapeutic community, occupational therapy) for schizophrenia in the psychiatric hospital ‘Sveti Ivan’. In outpatient services there is a lot of day hospitals offering psychoeducation, social skills training, family therapy, metacognitive training, psychodynamic therapy. On the other hand, a small number of patients are treated in CMHCs. In private practice, these types of treatment are delivered very rarely.

In Greece, ergotherapy starts during hospitalization whereas family therapy and psychoeducation start after discharge. Outpatient services include non-pharmacological treatment and it is offered by: University Mental Health Research Institute, Center for Psychotherapies, and Day Care Hospital. Many psychiatrists offer non-pharmacological treatments as part of their private practice. There are two relevant programs at the First Department of Psychiatry of the University of Athens – ‘Family Therapy’ (individual and multi-family therapy) and ‘Psychoeducation Programme’.

In Kosovo, there is individual and group non-pharmacological treatment; however, a separate department for this type of treatment for schizophrenia does not exist neither in inpatient nor in outpatient service. In CMHCs, they are offered in both individual and group form. In private practice, this type of treatment is offered only in individual form.

In Moldova, there are two of such services – the Department of Psychiatry, Addictions, Clinical Psychology, and Society of Psychiatrists, Addictologists, Psychother-apists, and Clinical Psychologists (AO SPNPPC). Inpatient service includes Department of Psychiatry where non-pharmacological treatments are mostly offered by clinical psychologists. They are planned during the case management. In CMHCs, they are delivered by psychologists and social workers, mostly in the individual form. In rural areas, mobile teams serve as facilitators of their delivery. In private practice, they are delivered by psychologists (CBT, psychoeducation, and Neuro-Linguistic Programming – NLP).

There is no institution specialized particularly for non-pharmacological treatment for psychotic disorders in Montenegro. Inpatient services, delivered at Psychiatric Clinic, include family and psychoanalytical therapy and therapeutic community meetings. Outpatient clinic within the Psychiatric Clinic as well as nine CMHCs widely distributed across the country offer family and psychoanalytical therapy. A small number of mental health professionals deliver non-pharmacological treatment to people with schizophrenia in their private practice.

Non-pharmacological treatment in both individual and group form is offered by inpatient service which is Day Hospital of University Clinic of Psychiatry in North Macedonia. There are seven CMHCs where these types of treatment are delivered in individual and group form. Non-pharmacological treatment for schizophrenia are minor part of treatment in private practice settings.

Psychologists, in collaboration with psychiatrists, offer support to people with schizophrenia in inpatient services in Romania. In outpatient services, psychologist offer some non-pharmacological treatments; however, without a department of psychotherapy. Psychiatrists and patients can freely choose various types of treatment with psychotherapists who have their own private practice. The expert from this country explained that non-pharmacological treatment is associated with personal desire and resources, and the state does not offer any training of this type for mental health specialists.

In Serbia, non-pharmacological treatment in inpatient services is offered by day hospitals belonging to university psychiatric institutions. There are two leading community mental health services in the country offering non-pharmacological treatment. In addition, there are CMHCs in other large cities that offer non-pharmacological treatment. However, the expert from Serbia was not sure that there are services specialized in non-pharmacological treatment for schizophrenia and, if they exist, there are a few of them. In private practice, non-pharmacological treatment is delivered to people with schizophrenia. Training in non-pharmacological treatment is usually organized by NGOs.

Lastly, for Slovenia, there is a ward specializing in the non-pharmacological treatment of people with schizophrenia at the University Psychiatric Hospital. It offers psychoeducation, supportive and social skills groups, CBT, psychodynamic therapy, etc. There is a small number of beds (10) available for this profile of patients. Group therapy is more common than individual therapy. All six psychiatric hospitals provide outpatient treatment and this type of treatment is also available in dispensaries across the country, in both individual and group form. A network of private outpatient services are mostly covered by health insurance and delivered in individual form. Community services include NGOs and social work centers which offer employment/vocational rehabilitation, educational programs, day centers, group homes and relative groups.

The type of services providing non-pharmacological treatment to people with schizophrenia is mostly public institutions (both inpatient and outpatient). Day hospitals offer this kind of intervention mostly through therapeutic community meetings (in Bulgaria, Croatia, Montenegro, North Macedonia, and Serbia). Therefore, these are mostly group forms of non-pharmacological treatment. However, individual forms are also provided, if needed, to some persons with severe mental illness. It seems that daily hospitals are part of both inpatient and outpatient services. Ambulatory treatment is another modality of providing such care within outpatient services. CMHCs are the third type of services which deliver non-pharmacological treatment to a greater or lesser extent to people diagnosed with schizophrenia in Albania, Croatia, Moldova, Montenegro, North Macedonia, and Romania. Another type of outpatient services that was identified is Center for Psychotherapies in Greece.

In general, profiles of mental health care professionals who deliver non-pharmacological treatment to people with severe mental illness include psychiatrists, (clinical) psychologists, psychotherapists, occupational therapists, and/or social workers. Non-pharmacological treatments usually delivered to people with schizophrenia in the services mentioned above are psychoeducation, supportive therapy, social skills training, family therapy, psychodynamic therapy, art therapy, occupational therapy, CBT, employment/vocational rehabilitation, and NLP.

## Discussion

In most SEE countries analyzed in this study the estimated percentage of people with schizophrenia who receive non-pharmacological treatments was below 40%. The non-pharmacological treatment for schizophrenia is part of inpatient care in all of these countries. The provision of non-pharmacological treatment is significantly more variable in outpatient statutory services and private services. Little information is available about the provision of non-pharmacological treatment in third/voluntary sector.

The explanations for the low availability of non-pharmacological treatments as identified by experts in our study mostly include: lack of human and financial resources, lack of training for clinicians, and pharmacotherapy dominance in the treatment for schizophrenia.

Some experts highlighted a lack of human and financial resources for the delivery of non-pharmacological treatment. This barrier has already been identified in published literature (e.g. Shafran et al., 2009). For example, in B&H, after the 1991 to 1995 war, economic situation remained instable. Additionally, one of the main issues in this country is the emigration of professionals to more prosperous countries, making human resources insufficient in various sectors (e.g. Sergi et al., 2004). Montenegro is still in the process of development of its mental health infrastructure due to distribution of financial resources to other economy sectors that were prioritized. More financial resources are needed for improving mental health care system in this country, as pointed out in the *Strategy for the protection and improvement of mental health in Montenegro (*2019*–2023)* (Ministry of Health of Montenegro, 2018). Serbia is also going through social transition, which slows down the process of implementing mental health strategies and action plans (Lecic Tosevski et al., 2010).

In Kosovo, for example, mental healthcare services are far away from the place of living of most patients because mental health centers (hospital units and CMHCs) are situated in big cities. Thus, care closer to home (CCTH) is needed. In this country, a lack of coordination between mental health services was also identified as an issue that inhibits the adequate delivery of non-pharmacological treatment to people with schizophrenia. Maybe this finding could be explained by the lack of human resources (especially psychiatrists in rural areas) and probably insufficient financial support for improving the collaboration between various mental health care services. A limited mental health care infrastructure and struggling with economical situation are two barriers underlined by Salize et al. (2014) as well.

In Croatia, a small number of mental healthcare services provide non-pharmacological treatment to people with schizophrenia. Interestingly, Croatia and Slovenia, offer specialist psychotherapeutic inpatient services for people with schizophrenia. They are, however, located in capital cities, thus indicating limited coverage. Established in 2017, in Croatia exist mobile teams that provide home visits (Stimac et al., 2018) which could overcome the aforementioned issue to some extent.

In Montenegro, the delivery of non-pharmacological treatment as part of private practice is not clearly and entirely regulated by the law. Hence, there is a gap between public and private practice. On the other hand, the National Health Insurance Fund contains non-pharmacological interventions as part of their official services included in their pricelist (Health Insurance Fund of Montenegro, 2015).

In Bulgaria, the National Health Insurance Fund does not support non-pharmacological treatments. Consequently, people with schizophrenia have to finance this kind of service on their own. This might be the reason why the estimated percentage of those who receive non-pharmacological treatment is around 20%. Other reasons could be financial and staff issues as well as a lack of joint strategic planning and coordination (European Psychiatric Association, 2018).

In Moldova the lack of human resources was underlined and this could explain heavy workload of clinicians, that was also highlighted by their expert. A survey conducted by De Vetten-Mc Mahon et al. (2019) identified workforce issues as one of the major problems with mental health care system in this country.

Inadequate or lack of training is also identified as one of the critical problems (e.g. in Serbia and Romania), which could be also said for other analyzed SEE countries. Hence, their public institutions could organize or at least initiate this type of training for mental health professionals as part of their professional development. For example, training in Serbia could be organized within the framework of *Action plan for implementation of the mental health program for the period from* 2019 *to 2026* (Ministry of Health of Serbia, 2018). One of the planned activities outlined in this document includes improvement/enrichment of the process of continuous education for mental health professionals. This might be a good way of increasing capacities for delivering non-pharmacological treatment to people with schizophrenia.

The finding that mostly public inpatient and outpatient services provide non-pharmacological treatment to persons with schizophrenia is similar to that reported by Injac Stevović et al. (2018) in the report covering 5 out of 12 SEE countries.

In private practice, usually, a small number of clinicians deliver non-pharmacological treatment to people with that category of disorders. There are some cases where these types of treatment are offered by NGOs or in collaboration with it, as reported by the experts from B&H and Slovenia.

Some of the non-pharmacological treatments listed by the experts as applied in their countries have been proved to be useful for people with schizophrenia, for example, social skills training, CBT, and family psychoeducation (Lecomte et al., 2014), as well as vocational rehabilitation (Twamley et al., 2003). However, some of these non-pharmacological treatments have not proved to be evidence-based approaches to the treatment of severe mental illness: psychodynamic therapy (Malmberg et al., 2001), art therapy (Crawford et al., 2012), and NLP (Witkowski, 2010).

Strengths of the present study are: exploring an under-researched topic, the inclusion of all countries typically considered in the SEE region; all types of statutory and non-statutory services were explored in the expert survey, due to the use of open-ended questions, and the experts had an opportunity to expand upon their answers.

The limitations of the present study could be summarized as follows: (1) the convenience sampling of mental health experts was used in the study, (2) the sample of experts and their institutions was small, including one expert per country, (3) the experts could have possibly been faced with lack of knowledge/data regarding some aspects of the healthcare services in their countries, although they were experts in the treatment of people with schizophrenia and were advised to consult other relevant experts in their respective countries when completing the expert survey, and (4) all participants were psychiatrists (a possible justification for this could be that their professional role is more dominant in the provision of care to people with schizophrenia compared to e.g. psychologists, especially in countries with strong medication-focused treatment approach).

## Conclusions

In the analyzed countries, non-pharmacological treatment for schizophrenia is part of inpatient services and it seems that the delivery of non-pharmacological treatment is more variable in outpatient services and private practice. However, information available about the delivery of non-pharmacological treatment in third/voluntary sector is scarce.

A contemporary, comprehensive treatment approach to severe mental illness should consider implementing non-pharmacological interventions (e.g. CBT, family therapy, psychoeducation, and supported employment) to a greater extent. In most SEE countries, human and/or financial resources to deliver non-pharmacological treatment for schizophrenia are scarce. One way to overcome these barriers would be to implement generic, evidence-based interventions that doesn’t require long training of staff and that could be delivered by wide range of professionals. This recommendation is supported by the notion of Andreou and Moritz (2016) that low-cost and short interventions are suitable for situations/conditions where resources are limited. Furthermore, better coordination between mental health care services and clear legislation regarding delivering non-pharmacological interventions to people with schizophrenia are needed. Additionally, it is important to enable the equal geographical spread of community mental health centers (CMHCs) and other community-based mental health care services (e.g. NGOs such as user-led organizations).

## Appendix

### Expert survey

A. GENERAL INFORMATION ABOUT THE EXPERT COMPLETING THIS SURVEY:
1. Your name: _______________
2. Country: ________________
3. Institution: ___________________
4. Gender: _____________
5. Age: _____________________
6. Profession: ___________________
7. Years of professional experience in the field of mental health: _______________
B. MAIN QUESTIONS
8. How many patients with schizophrenia receive psychosocial interventions/psychotherapy in your country?
Please express your estimate in percentages: ____________ %.
9. If you think that the percentage you indicated is low, could you please elaborate the reasons you believe are behind such an estimate: ______________________________
10. Is there a service/expert in your country which specialises in psychotherapy/psychological therapy for schizophrenia, please provide details: ________________________________________
11. Can you please specify which settings/services offer psychosocial interventions/ psychotherapies in your country?
(a) Inpatient services YES/NO

*Please describe briefly how are psychosocial interventions/psychotherapies for people with schizophrenia implemented in inpatient services, for example, do you have a separate Department for such interventions, do you have special time slots where you apply them, do you offer psychosocial interventions/psychotherapies in individual or group form, etc.)?*

______________________________________________

(b) Outpatient services YES/NO (including Community Mental Health Centres)

*Please describe briefly how are psychosocial interventions/psychotherapies for people with schizophrenia implemented in outpatient services, for example, do you have a separate Department for such interventions, do you have special time slots where you apply them, do you offer psychosocial interventions/psychotherapies in individual or group form, etc.)?*

______________________________________________

(c) Private practice YES/NO

______________________________________________

(d) Other (please specify) ______________________________________________
